# Mixture Toxicity of Bensulfuron-Methyl and Acetochlor to Red Swamp Crayfish (*Procambarus clarkii*): Behavioral, Morphological and Histological Effects

**DOI:** 10.3390/ijerph14121466

**Published:** 2017-11-27

**Authors:** Jixin Yu, Elvis Genbo Xu, Yan Ren, Shiyu Jin, Tanglin Zhang, Jiashou Liu, Zhongjie Li

**Affiliations:** 1 State Key Laboratory of Freshwater Ecology and Biotechnology, Institute of Hydrobiology, Chinese Academy of Sciences, Wuhan 430072, China; jxyu001@126.com (J.Y.); renyan@ihb.ac.cn (Y.R.); jinshiyu@ihb.ac.cn (S.J.); jsliu@ihb.ac.cn (J.L.); zhongjie@ihb.ac.cn (Z.L.); 2 College of Life Sciences, University of Chinese Academy of Sciences, Beijing 100049, China; 3 Department of Environmental Sciences, University of California, Riverside, CA 92521, USA; genboxu@ucr.edu

**Keywords:** *Procambarus clarkii*, mixture of bensulfuron-methyl and acetochlor, acute toxicity, LC_50_, behavior, histopathology, rice herbicide

## Abstract

The mixture of bensulfuron-methyl and acetochlor (MBA) has been widely applied as a rice herbicide in China, but the mixture toxicity of MBA to aquatic organisms is largely unknown. The current study aims to investigate the acute effects of MBA to juvenile red swamp crayfish, *Procambarus clarkii*. Firstly, a 96 h semi-static exposure was conducted to determine the Lethal Concentration 50 (LC_50_) values at 24, 48, 72 and 96 h, as well as to assess the behavioral and morphological effects. A second 96 h exposure was conducted at an MBA concentration of 50% of the 96 h LC_50_ (72.62 mg/L) to assess the histological changes in the gill, perigastric organ, muscle, heart, stomach, and midgut. The results showed that MBA exhibited low acute toxicity with the 24, 48, 72 and 96 h LC_50_ values of 191.25 (179.37–215.75), 166.81 (159.49–176.55), 154.30 (148.36–160.59) and 145.24 (138.94–151.27) mg/L, respectively. MBA-exposed crayfish showed body jerk, belly arch, equilibrium loss, body and appendage sway, and lethargy; and the dead crayfish showed dark gray or grayish-white body color and separated cephalothorax and abdomen. At 72.62 mg/L, MBA exposure caused significant histopathological alterations, mainly including the cuticular and epithelial degeneration of all the gills; atrophy of tubule lumina and cellular vacuolation of the perigastric organs (61.15 ± 9.90% of the tubules showed lesions); epithelial hyperplasia (48.40 ± 9.00%), myocardial fibers and epithelial cell lysis (17.30 ± 2.01%), and hemocytic infiltration of the hearts; cuticular swelling (15.82 ± 2.98%) and vacuolate connective tissue (11.30 ± 2.47%) of the stomachs; atrophied bladder cell and fragmented longitudinal muscles (95.23 ± 4.77%) of the midguts; and slight myofibers fragmentation and lysis (7.37 ± 0.53%) of the abdominal muscles. Our results indicate that MBA can cause behavioral, morphological and histopathological effects on juvenile *P. clarkii* at relatively high concentrations, but its acute toxicity is low compared with many other common herbicides.

## 1. Introduction

Pesticides have long been attracted public concerns for their potential environmental risks. When released into the soil, water, and atmosphere, pesticides can affect the health of non-target organisms, including human beings in different ways, such as direct contact, transformation and migration via food webs [1,2,3]. Pesticides have been considered as a major pollutant to aquatic ecosystems [4,5].

Bensulfuron-methyl is one of the most commonly used rice herbicides in the world [6]. Concentrations of this herbicide in Japan were detected from 0.1 to 2.3 μg/L in river and lake water [7], and from <0.01 to 139.97 μg/L in water of rice paddy, its drainage channel and downstream after a bensulfuron-methyl usage of 51 g/hm^2^ over 28 days [8]. Bensulfuron-methyl has been shown to cause genotoxicity and inhibit embryonic development in zebrafish *Danio rerio* [6,9]. The 96 h Lethal Concentration 50 (LC_50_) values of bensulfuron-methyl to five freshwater algae ranged from 0.015 to 6.20 mg/L [10,11]. Acetochlor is another globally used herbicide, which has also been widely applied in China [12]. Previous studies on acetochlor demonstrated concentrations of 0.05–3.32 μg/L in surface water in the USA [13,14]; 0.47–11.76 μg/kg in sediments and 0.03–709.37 μg/kg in riparian soils of the Songhua River Basin in China [15]; and 2.0 μg/L in water, 3.9–6.6 μg/g in sediments and 3.3–11.7 μg/g in fish of Huaihe River in China [16]. A low concentration of acetochlor (≤0.3 mg/L) caused endocrine disruption of the thyroid system of zebrafish *D. rerio* larvae [17]. Li et al. [9] reported that environmentally relevant concentrations (0.02–2 μg/L) of acetochlor affected larval development and adult brain of rare minnow *Gobiocypris rarus*. It also caused a dose-dependent oxidative stress and DNA damage in livers of tadpole Mongolian toad *Bufo raddei* (0–0.068 mg/L) [18]. Due to its potential carcinogenicity and genotoxicity, the U.S. EPA has classified acetochlor as a probable human carcinogen [19].

Bensulfuron-methyl has specific efficacy in controlling broadleaf weeds and sedges, and acetochlor has specific efficacy in controlling annual gramineous weeds, which are tolerant to bensulfuron-methyl [20]. Therefore, bensulfuron-methyl and acetochlor are commonly mixed as a mixture herbicide (MBA). Combination of herbicides may introduce additive effects of toxicity [21]. As mentioned above, the toxicity of bensulfuron-methyl and acetochlor has been individually investigated, but the mixture effects of MBA to aquatic organisms are still unknown.

The red swamp crayfish *Procambarus clarkii* is a widely distributed freshwater species, which lives in lakes, rivers, ponds, rice fields, ditches, irrigation systems and marshes [1]. Its strong tolerance to environmental variables and high fecundity make *P. clarkii* an easy dominant and key species of the habitat, and thus has significant impacts on the primary productivity, food webs, water quality, sediments, and biodiversity of the habitat [1,22,23]. In addition, *P. clarkii* has become a globally important aquaculture species with an annual yield of more than 700,000 tons in recent years, which holds the highest yield share of freshwater crayfish [23]. In China, America, and Portugal, crayfish aquaculture is often integrated with rice planting as a rice-crayfish integrated system (RCIS) [24,25,26]. The applied MBA in RCIS may affect the health of crayfish as well as human consuming polluted crayfish. MBA may also enter waterbody outside the RCIS through drainage, migration of spray, surface runoff, groundwater seepage, atmospheric deposition, and accidental MBA spill.

*P. clarkii* is an ideal model and has been used in numerous toxicology studies [22,24,26,27,28]. It has an “open” circulatory system, with internal organs directly immersed in hemolymph. The internal organs of crayfish are therefore in direct and constant exposure to toxicants introduced into the crayfish hemolymph via gills absorption from water [29]. *P. clarkii* is benthic and omnivorous [22], so it may be readily and consistently exposed to MBA via direct contact and feeding on MBA-contaminated sediment.

This study aimed to assess: (1) the acute 96 h toxicity of MBA (0, 115, 130, 145, 160, 175, and 190 mg/L) to *P. clarkii* and determine the LC_50_ at 24, 48, 72 and 96 h; (2) the morphological and behavioral effects; and (3) histological changes in the gill, perigastric organ, muscle, heart, stomach, and midgut at a lower concentration (50% of the 96 h LC_50_) of MBA. The results of this study provide a better understanding of the mixture toxicity of MBA to *P. clarkii*, which are also useful to guide the application of MBA in RCIS.

## 2. Materials and Methods

### 2.1. Test Organisms and Chemicals

Juvenile *P. clarkii* were selected and bought from a crayfish breed cooperative in Qianjiang City, Hubei Province, China. Perforated plastic containers with wet aquatic plants were used to transport the crayfish to reduce death and cannibalism. Crayfish were transported to the laboratory of Institute of Hydrobiology, Chinese Academy of Sciences, within 2 h, and then were adapted in a 0.5 cm deep tap water tank for 2 h. Crayfish were acclimatized to the test conditions (i.e., 20 °C and a 16:8 h light:dark photoperiod) over two weeks. Crayfish were fed with pellet foods (28% crude protein, 4% crude lipid, 8% crude fiber) every 6 days during the acclimation until 48 h prior to the test. To minimize natural aggression and cannibalism of the crayfish, polyvinyl chloride pipes and artificial aquatic plants were placed in the containers during the acclimation and test phase. Less than 5% mortality was observed during the acclimation. Healthy intermolt-staged juveniles with complete appendages and chelipeds were selected (mean weight of 0.27 ± 0.05 g).

Technical-grade bensulfuron-methyl (99.60%) and acetochlor (99.0%) (Beijing JSYH Chemical Technology Research Institute, Beijing, China) were mixed as MBA, with a ratio of 1:2 (most commonly applied in commercial MBA). MBA was then dissolved in double-distilled water as MBA solution.

### 2.2. Acute Toxicity Tests

Preliminary range-finding tests (5–200 mg/L) were initially performed to define the testing concentrations. Then 210 randomly selected crayfish were exposed to six MBA concentrations (115, 130, 145, 160, 175, 190 mg/L) and water control, with 10 individuals in one 20 L glass exposure chamber in triplicate (*N* = 21). The test solution was daily renewed to maintain the test concentrations. No feed was performed during the test. Mortality and sublethal effects including abnormal behavior, spastic movement, lethargy, and morphological changes were recorded at 1, 12, 24, 48, 72 and 96 h of exposure. Death was defined as the immobility of a crayfish when probed gently with a glass rod within 5 min [26]. Tap water used in the acclimation and exposure was aerated for 48 hours to remove chlorine, and then ultraviolet sterilized. Water temperature and photoperiod were set at 20 °C and a 16:8 h light:dark photoperiod, respectively. Water quality parameters were daily measured, and the mean of temperature, dissolved oxygen, total hardness, pH and ammonia were 20 ± 0.6 °C, 7.6 ± 0.6 mg/L, 127 ± 9 mg/L (CaCO_3_), 7.50 ± 0.36 and <0.1 mg/L, respectively.

### 2.3. Histopathology

A second 96 h exposure was carried out for histopathology assessment with 60 crayfish. The crayfish were exposed to a water control and 72.62 mg/L MBA concentration (50% of the 96 h LC_50_ determined by the above-mentioned acute toxicity test in triplicate) (*N* = 6). The exposure conditions were the same as the acute toxicity test. At 96 h, fresh gills, perigastric organs, abdominal muscles, hearts, stomachs, and midguts of 12 living crayfish in both the treatment and control groups were dissected. The tissue samples were fixed in Bouin’s Solution, dehydrated, and then processed for paraffin wax embedding. The embedded tissues were cut into 4 μm sections and stained with hematoxylin and eosin (H&E) [30]. The sections of the paraffin blocks were imaged with an OLYMPUS BX53 microscope (Olympus Corporation, Tokyo, Japan). The quantitative histological effects were tested as much as possible by measuring the percentages of damaged area or number, with three valid sections as repeats (*N* = 3). All of the rounded and elliptical gill crosscuts, and those with one or multiple obvious lesions among these gill crosscuts were counted one by one, respectively; similar method was performed on tubules in the perigastric organs. For the hearts, stomachs, midguts and abdominal muscles, areas of the total samples and the parts with different lesions were measured, respectively, and percentages of the damaged muscles in the midguts was the ratio of the area of the muscles with lesions to all the muscles.

### 2.4. Statistical Analysis

Data analyses were performed in SPSS, version 13.0 (Armonk City, NY, USA). The mortalities at 24, 48, 72 and 96 h of the treat groups were analyzed with Probit analysis to determine the 24, 48, 72 and 96 h LC_50_ and 95% confidence limits. The mortality in each chamber was the ratio of the number of dead crayfish to 10, and the mortalities used for LC_50_ determination were the mean mortalities of the three repeats of each MBA concentration. In the Probit analysis, the mortalities were converted to probits and the MBA concentrations were log-transformed. For analysis of the histological changes, percentages or transformed percentages of lesions were tested for normality first. Herein, data on the damaged muscles of midguts was analyzed with Mann–Whitney *U* test and the other data with independent-sample *T* test. *p* < 0.05 was considered to be significantly different. Mortalities in the regressions and percentages of lesions were presented as the mean ± standard error (Mean ± SE; *N* = 3).

## 3. Results

### 3.1. Descriptive Behavioral and Morphological Effects

At the start of the exposure, crayfish moved fast and erratically with body jerk and hyperexcitability, and some crayfish arched their belly. Then, their movement slowed down, and they started to roll and fall to the bottom of the tank due to equilibrium loss. Before their death, antenna and appendages of some carotic crayfish still swayed slowly, and some showed a rapid tail-flip when touched. The body color of the MBA-exposed crayfish became dark gray or grayish-white. The cephalothorax and abdomen of dead individuals were separated.

### 3.2. Lethal Concentration 50 (LC_50_) of MBA

**Figure 1:**
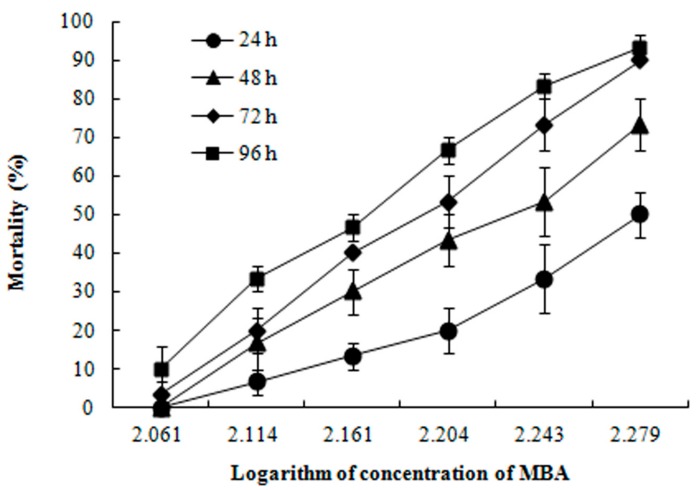
Mortality of juvenile *P. clarkii* exposed to mixture of bensulfuron-methyl and acetochlor (MBA) at 24, 48, 72 and 96 h (Mean ± SE). No death was noted in the water control group.

No mortality was observed in the control groups within the 96 h. The concentration- and time-dependent trends of mortalities of juvenile *P. clarkii* after MBA exposure are shown in Figure 1. The LC_50_ of MBA were 191.25, 166.81, 154.30 and 145.24 mg/L at 24, 48, 72 and 96 h, respectively (Table 1).

### 3.3. Histopathological Effects of MBA

#### 3.3.1. Gills

**Figure 2:**
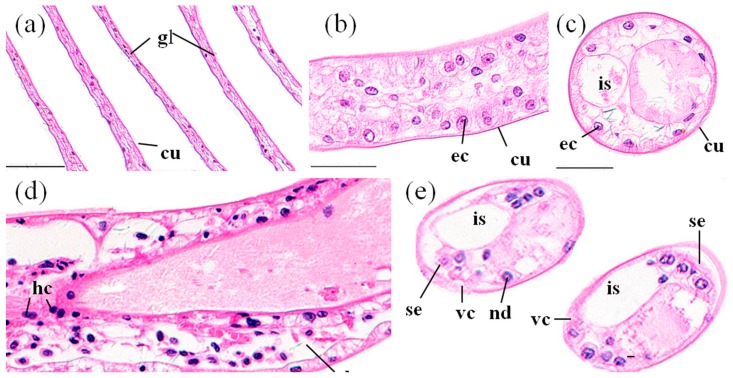
The gills of *P. clarkii* after 96 h exposure to: a control solution (**a**–**c**); and 72.62 mg/L MBA (**d**,**e**). (**a**) Control gill showed uniform gill lamella (gl) and cuticula (cu) with clear structure (200×). (**b**) Control gill lamella showed uniform arrangement of the gill epithelial cells (ec) (400×). (**c**) Control gill lamella showed uniform intralamellar space (is) (400×). (**d**) Damaged gill lamella exhibited granular hemocytes (hc) inside the intralamellar space, some degeneration in the cuticula (dc) and some disorganization of the gill epithelial cells (de) (400×). (**e**) Damaged gill lamella exhibited some vagueness of the cuticula (vc), swelling and darkening of nucleus of the gill epithelial cells (nd), and separation of some gill epithelial cells from the cuticula (se) (400×). cu, cuticula; ec, epithelial cell; is, intralamellar space. H&E stain, scale bars = 100 μm.

The gill of *P. clarkii* is divided into a central axis and branching gill filaments (lamellae) (Figure 2a). Each gill filament is limited by a single epithelial layer and covered with a thick cuticula (Figure 2b,c). In the control groups, the gills showed uniform arrangements of lamellae and intralamellar spaces with clear cuticula (Figure 2a,c). Under the cuticula, epithelial cells are closely and uniformly located (Figure 2b,c). After 96 h of MBA exposure, almost all of the gills exhibited granular hemocytes inside the intralamellar space and disorganization of the epithelial cells (Figure 2d). The vagueness of the cuticula, heavier staining of nucleus of the epithelial cells and separation of some epithelial cells from the cuticula were also noted (Figure 2e). The ratio of damaged gills in the MBA-treated crayfish (100%) was significantly higher than that in the control crayfish (3.73 ± 1.10%) (*p* < 0.001, Figure 8).

#### 3.3.2. Perigastric Organs

**Figure 3:**
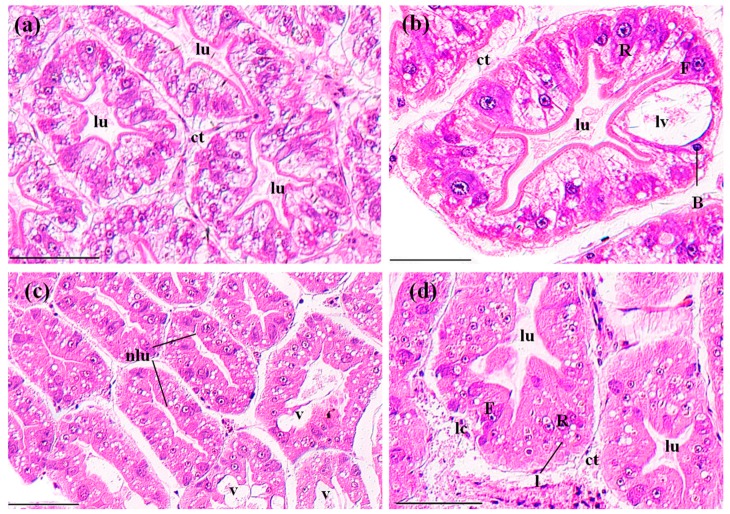
The tubules in perigastric organs of *P. clarkii* after 96 h exposure to: a control solution (**a**,**b**); and 72.62 mg/L MBA (**c**,**d**). (**a**) Control tubules with normal structures and intertubular connective tissue (ct) (200×). (**b**) Control tubules showed different cell types, including B-cell (B), F-cell (F) and R-cell (R). Note the large vacuole (lv) which occupies most of the B-cell (400×). (**c**) Damaged tubules exhibited narrowing of the lumina (nlu) and vacuolation (v) in some cells (200×). (**d**) Damaged tubules exhibited slight cellular destruction and lysis of the cytoplasm (lc) in some cells (400×). Note the decrease of the lipid granules in (**c**,**d**). lu, lumen; ct, connective tissue; B, B-cell; F, F-cell; R, R-cell; l, lipid granules. H&E stain, scale bars = 100 μm.

Perigastric organs are composed of numerous blind ended tubules that are separated by connective tissues (Figure 3a). In the tubules, there are three types of epithelial cells, absorptive (R) cells, secretory (B) cells and fibrillar (F) cells, and a stellate lumen in the center (Figure 3b). In the control groups, the perigastric organs exhibited a clear arrangement of the epithelial cells and lumina (Figure 3a,b). In the MBA-treated perigastric organs, 61.15 ± 9.90% of the tubules showed lesions, which was significantly higher than the control perigastric organs (5.52 ± 0.96%) (*p* < 0.01, Figure 8). The main lesions included narrowing of the lumina and distinct vacuolation in some cells (Figure 3c), slight cellular destruction and lysis of the cytoplasm in a few tubules (Figure 3d).

#### 3.3.3. Hearts

**Figure 4:**
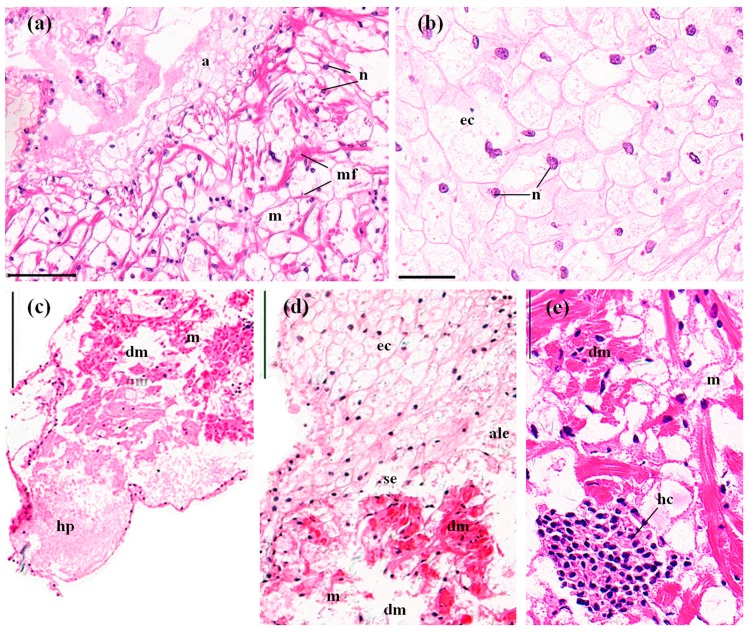
The hearts of *P. clarkii* after 96 h exposure to: a control solution (**a**,**b**); and 72.62 mg/L MBA (**c**–**e**). (**a**) Control tissues with normal structures of the myocardium (m) and adventitia (a). Note the multinucleated and branched myocardial fibers (mf) (200×). (**b**) Control adventitia tissues with uniform arrangement of the epithelial cells (ec). Note the netlike adventitia, the epithelial cells without cytoplasm, and the eccentrically located nuclei (n) (400×). (**c**) Damaged hearts exhibited hyperplasia of the epithelial tissue of the adventitia, and lysis and vacuolation in the myocardial fibers (dm) (100×). (**d**) Damaged hearts exhibited swelling, lysis and vacuolation in the myocardial fibers (dm), atrophy and lysis of the epithelial cells of the adventitia (ale), and slight separation of the adventitia from the myocardium (se). (200×). (**e**) Damaged hearts exhibited swelling of the myocardial fibers (dm) and hemocytic infiltration (hc) inside the myocardium (400×). myocardium (m); n, nuclei; ec, epithelial cell. H&E stain, scale bars = 100 μm.

The myocardium consists of multinucleated and branched myocardial cells. An adventitia (epicardium) invests the whole external surface of the myocardium (Figure 4a). The adventitia is composed of several layers of uninucleated epithelial cells, which vary in size and shape. Typically, there is no cytoplasm in these epithelial cells, and this gives the adventitia a netlike structure. The nuclei of the epithelial cells are eccentrically located (Figure 4b). The control crayfish exhibited normal and clear myocardial cells and adventitia, with no hyperplasia and 1.27 ± 0.35% of vacuoles (Figure 4a,b, Figure 8). Following the 96 h MBA exposure, significant hyperplasia of the epithelial tissue of the adventitia was observed in 48.40 ± 9.00% (*p* < 0.05) of the total section area (Figure 4c, Figure 8). The adventitia also exhibited some atrophy and lysis of the epithelial cells and slight separation from the myocardium (Figure 4d). Swelling, lysis, and vacuolization of myocardium and hemocytic infiltration inside the myocardium were also commonly detected (Figure 4c–e). In total, 17.30 ± 2.01% of the section area was vacuolar, which was significantly higher than that in the control hearts (*p* < 0.01, Figure 8).

#### 3.3.4. Stomachs

**Figure 5:**
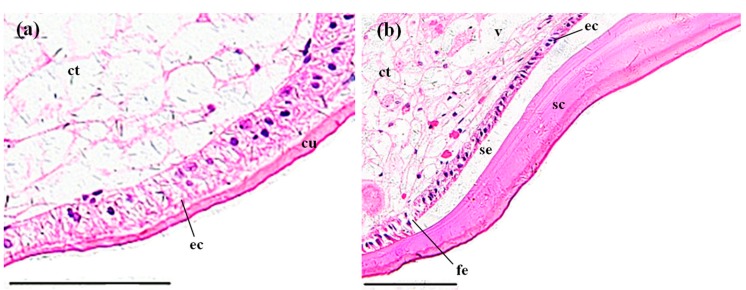
The stomachs of *P. clarkii* after 96 h exposure to: A control solution and 72.62 mg/L MBA. (**a**) Control stomachs showed normal cuticula (cu), epithelial cells (ec) and connective tissues (ct) with clear and uniform structure (200×). (**b**) Damaged stomachs exhibited swelling of the cuticula (sc), fragmentation of the epithelial layer (fe), separation of the epithelial layer from the cuticula (se) and some vacuolization of the connective tissues (v) (200×). ec, epithelial cell. H&E stain, scale bars = 100 μm.

A normal stomach is covered with a thick cuticula which is underlain by a single epithelium that composed of simple columnar epithelial cells. In contact with the epithelium is a thick connective tissue layer (Figure 5a). No obvious abnormal-structures or lesions were found in the control stomachs (Figure 5a). In the MBA-treated stomachs, the cuticula became thicker, with a significantly higher ratio (15.82 ± 2.98%) than the control groups (0.21 ± 0.10%) (*p* < 0.05, Figure 8); the epithelium became fractured and detached from the cuticula, and some of the connective tissues became vacuolated (Figure 5b), with a significantly higher share (11.30 ± 2.47%) of the section area than that of the control stomachs (0.45 ± 0.02%) (*p* < 0.05, Figure 8).

#### 3.3.5. Midguts

**Figure 6:**
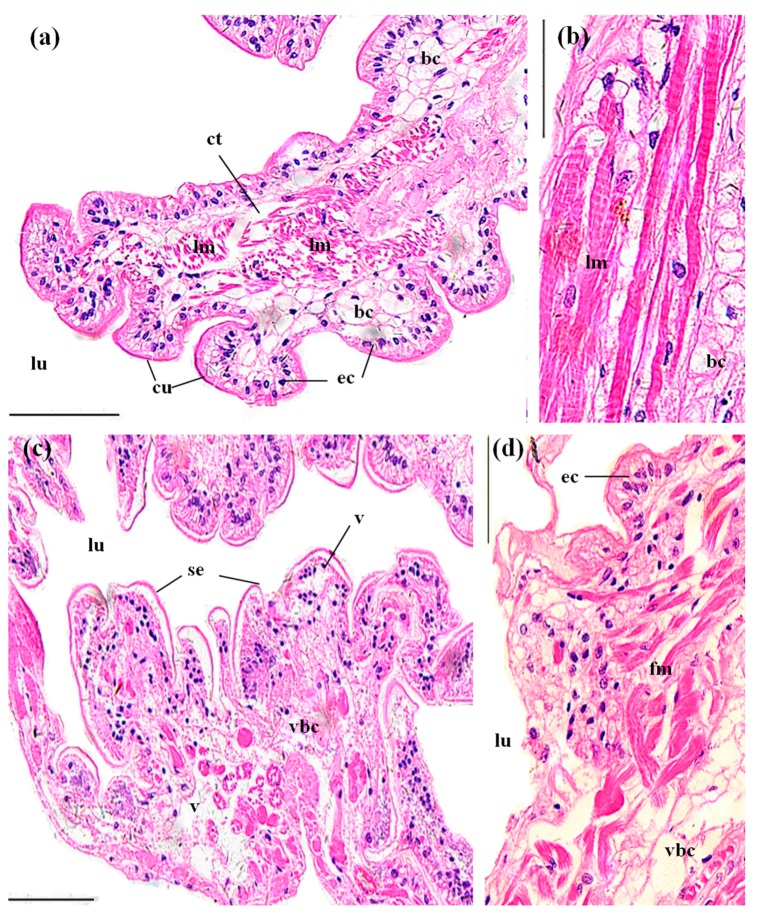
The midguts of *P. clarkii* after 96 h exposure to: a control solution (**a**,**b**); and 72.62 mg/L MBA (**c**,**d**). (**a**) Control midguts with uniform arrangement of the cuticula (cu), epithelial cells (ec), bladder cells (bc) and longitudinal muscles (lm) (200×). ct: connective tissues. (**b**) Control midguts with uniform arrangement of the longitudinal muscles (lm) and the bladder cells (bc) (400×). (**c**) Damaged midguts exhibited separation of the epithelium from the cuticula (se), vagueness (vbc) and lysis, and vacuolation in some bladder cells (v) (200×). (**d**) Damaged midguts exhibited some vagueness of the bladder cells (vbc) and fracture of the longitudinal muscles (fm) (400×). ec, epithelial cell; lu, lumen. H&E stain, scale bars = 100 μm.

The midgut of *P. clarkii* is composed of several longitudinal ridges with cuticula. Each ridge consists of a simple columnar epithelium with a fibrous cytoplasm and mottled nuclei, and subepithelial connective tissues that consist of bladder cells and longitudinal muscles. The bladder cells are massive and the vacuolate cells have peripheral ovoid nuclei (Figure 6a,b). The midguts of the control crayfish showed normal and clear structure of the cuticula, epithelial cells, bladder cells and longitudinal muscles (Figure 6a,b). In the MBA-treated midguts, the epithelium was detached from the cuticula (Figure 6c). The bladder cell became significantly damaged (vagueness, lysis and vacuolation, 4.78 ± 1.28%) (Figure 6c,d) than that in the control midguts (*p* < 0.05, Figure 8), and 95.23 ± 4.77% of the longitudinal muscles became fractured (Figure 6d), which was significantly higher than that in the control groups (*p* < 0.05, Figure 8).

#### 3.3.6. Abdominal Muscles

**Figure 7:**
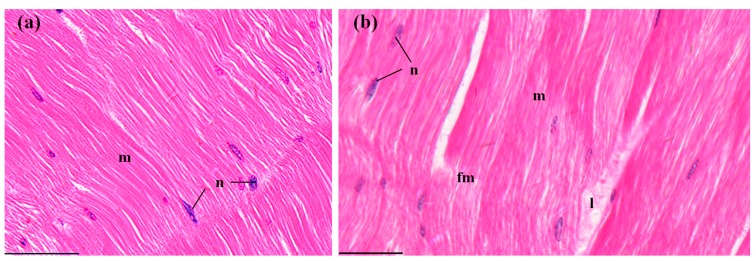
The muscles of *P. clarkii* after 96 h exposure to: A control solution and 72.62 mg/L MBA. (**a**) Control muscles with ordered myofibers (m) and oval nuclei (n) (400×). (**b**) Damaged muscles exhibited slight fracture (fm) and lysis (l) of some myofibers (400×). m, myofiber; n, nucleus. H&E stain, scale bars = 100 μm.

**Figure 8:**
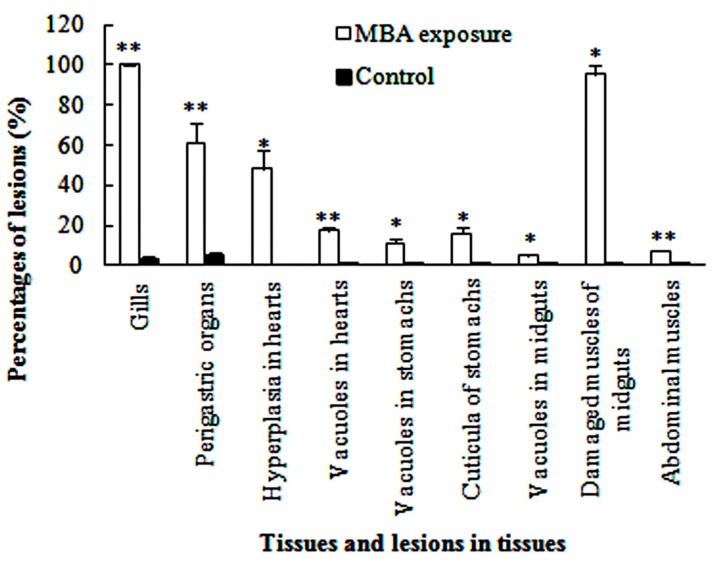
Percentages of the number or area of lesions in the six tissues of MBA-treated and control *P. clarkii* (Mean ± SE). Herein, all of the rounded and elliptical gill crosscuts, and those with one or multiple obvious lesions among these gill crosscuts were counted one by one, respectively; similar method was performed on tubules of the perigastric organs. For the hearts, stomachs, midguts and abdominal muscles, the areas of the total samples and parts with different lesions were measured, respectively, and only hyperplasia and vacuoles in the hearts, cuticula damages and vacuoles in the stomachs, and vacuoles and muscle damages in the midguts were separately analyzed for they were the main lesions in these organs. Percentages of the damaged muscles in the midguts were the ratio of the area of the muscles with lesions to all the muscles. Asterisks represent significant differences (* *p* < 0.05, ** *p* < 0.01).

The abdominal muscles of control crayfish exhibited compact and uniform myofibers with oval nuclei (Figure 7a). After MBA exposure, a generally intact structure with slight rupture and lysis of a few myofibers was observed (Figure 7b), but the ratio of lesions (7.37 ± 0.53%) was still significantly higher than that in the control groups (*p* < 0.001, Figure 8).

## 4. Discussion

### 4.1. Behavioral and Morphological Effects of MBA

Behavioral responses in contaminant-exposed crustaceans have been widely reported and summarized by Hebel et al. [29]. The incunabular irritation (e.g., rapid swimming, attempting to “escape”, and convulsion), equilibrium loss, and faint at later stages observed in the current study were similar to the observations in crustaceans exposed to etofenprox [3], terbufos [22], deltamethrin [26], and chlorpyrifos [31]. Increased aggression was also observed in etofenprox-exposed narrow-clawed crayfish *Astacus leptodactylus* [3]. In deed, the behavioral responses were also observed in our preliminary tests at lower concentrations. The abnormal behaviors at sublethal MBA concentrations may result in vulnerability to predation, decreased feeding success and growth [22]. These mean that MBA at relatively lower or environment-realistic concentrations can cause sublethal and long-term effects on juvenile *P. clarkii* and *P. clarkii* population. The change of body color is a common indicator of pesticide toxicity, as reported in pesticide-treated *P. clarkii* and oriental river prawn *Macrobrachium nipponense* [32,33].

### 4.2. Comparison of MBA 96 h LC_50_ with Other Herbicides

The 96 h LC_50_ is one of the most important parameters in acute toxicity assessment [34]. The 96 h LC_50_ value of MBA herein was higher than 100 mg/L, suggesting a low toxicity to juvenile *P. clarkii*. The 96 h LC_50_ value of MBA is 1–3 orders of magnitude higher than that of trifluralin, paraquat, propanil, molinate or thiobencarb, while similar to 2,4-D and two orders of magnitude lower than sulfometuron (Table 2). The acute toxicity of MBA to *P. clarkii* is much lower than many other herbicides.

### 4.3. Histopathological Effects of MBA

Bensulfuron-methyl and acetochlor are both lipophilic compounds, which enter aquatic animals by transferring across the body surface, notably the gills [38]. Gills are thus a primary target organ and may be the first organ to exhibit toxic symptoms [1]. Despite the relatively low acute toxicity of MBA, juvenile crayfish exhibited severe pathological alterations in gills after exposure to MBA at a concentration of 50% of the 96 h LC_50_. Some pathological changes, such as granular hemocytes inside the intralamellar space, disorganization of the epithelial cells, vagueness of the cuticula, and separation of some epithelial cells from the cuticula, were also observed in the gills of trichlorfon, chlorpyrifos, ethion and etofenprox exposed crustaceans [1,3,31,39]. A study of another sulfonylurea herbicide, metsulfuron-methyl, suggests that the gills of Nile tilapia *Oreochromis niloticus* exhibits a higher specific activity than other organs, indicating that gill is the major organ in absorbing metsulfuron-methyl [40]. The damaged gills of aquatic animals will further result in compromised oxygen consumption and osmoregulation. As an example, both the histological changes of gills and disruption of osmoregulatory processes of shrimp *Penaeus japonicus* were noted after exposure to sublethal or lethal concentrations of fenitrothion [41].

Perigastric organ is a major metabolic organ of crayfish, which functions digestion, absorption, secretion, and excretion. Perigastric organ is also a major detoxification organ of crustacean and is sensitive to pesticides, metals and other water-borne pollutants [42,43,44]. Acetochlor-induced histopathological injuries in livers or perigastric organs have been reported in other animals. In rat *B. raddei*, for example, acetochlor was found to induce oxidative stress and DNA damages in livers at <0.068 mg/L [18], and caused hepatic cell necrosis and inhibition of glutathione levels at 2000 mg/kg [45]. The main pathological alterations of perigastric organs in this study, including atrophy of the lumina, vacuolation and cellular destruction, were also observed in perigastric organs of *P. clarkii* upon exposure to some organophosphorus pesticides [1,31,46]. These lesions were probably due to the accumulation of MBA or its metabolites in the cells of perigastric organs, or increased activity of the lysosomal enzymes which destroy cell organelles [1].

Epithelial hyperplasia of the adventitia, swelling and lysis of the myocardium was observed in hearts in this study, similar to a study on *P. clarkii* after chlorpyrifos exposure [31]. Such pathological changes in hearts may result in abnormal structures and functions of hearts. For example, distinct heart malformations were detected in perfluorooctanesulfonate-exposed zebrafish *D. rerio* [47], heart rates were also significantly inhibited in toxicant-exposed zebrafish *D. rerio* and toad *Bufo bufo gargarizans* [47,48,49]. The affected heart functions may further cause heart failure leading to the acute death of the MBA-exposed crayfish.

The pathological alterations in the stomachs and midguts of the MBA-exposed crayfish were also found in tiger prawn *Penaeus monodon* after triazophos exposure [50]. Similarly, after exposure to 1.5–3.0 μg/L fenvalerate for 96 h, atrophy and necrosis of the epithelial cells, exfoliation of the mucosal epithelium and lymphocytic infiltration into the lamina propria were detected in the intestines of mrigal *Cirrhinus mrigala* [30].

When exposed to herbicides, the fatty organs (e.g., liver, perigastric organ, ovary and visceral adipose) of aquatic animals tend to accumulate higher concentrations of residues than other organs such as muscle [51,52]. Freshwater crayfish abdominal muscle has low contents of lipids (less than 0.8% of dry matter) [23]. This may explain the lesser histological effects of MBA on muscles in this study. Several other investigators have also detected the lowest concentrations of toxicants in fish and crayfish muscles [52,53,54]. In addition, the hard carapace of crayfish may absorb a portion of toxicants and reduce the amount of toxicants entering muscles from the body surface. Ethion and its degradation products, for instance, exhibited higher concentrations in carapaces than muscles of *P. clarkii* [1]. Further study is needed to measure the residue concentrations of MBA in muscle tissues of *P. clarkii* to assess the human health risk.

### 4.4. Environmental Risk of MBA in RCIS

The trenches of RCIS in China usually account for ~10% of the total rice field area, with a water depth of 1–1.5 m [55], indicating the total water volume of RCIS is ≥10^8^ L/ha (total area × percentage of trench × water depth, i.e. 10^6^ m^2^/ha × 0.1 × 1 m = 10^5^ m^3^/ha = 10^8^ L/ha). The actual dose of commercial MBA (~15% active ingredient) on rice is approximately 750 g/ha (~112.5 g/ha of pure MBA). Concentrations of MBA in the water of RCIS are thus estimated as <1.125 μg/L (112.5 g/ha/10^8^ L/ha) and ~5 orders lower than its 96 h LC_50_, indicating that MBA in RCIS and its surrounding waterbodies poses a low acute risk to *P. clarkii*. However, as the lethal, behavioral and histopathological effects of MBA were detected in juvenile *P. clarkii* under relatively high concentrations, the need to further evaluate the long-term effects of MBA at environment-realistic concentrations including toxicological endpoints of high sensitivity such as genotoxicity and molecular toxicity is apparent.

## 5. Conclusions

This study shows that mixture of bensulfuron-methyl and acetochlor (MBA) is low toxic to juvenile *P. clarkii*, and its acute toxicity is low compared with many other common herbicides, but it can cause behavioral, morphological and histopathological effects (especially for gills, perigastric organs and hearts) on juveniles at relatively high level.

## Figures and Tables

**Table 1:** Regression equations, Lethal Concentration 50 (LC_50_) values, and 95% confidence limits of mixture of bensulfuron-methyl and acetochlor (MBA) to juvenile *P. clarkii* at 24, 48, 72 and 96 h.

Time (h)	Regression Equation	R^2^	LC_50_ (mg/L)	95% Confidence Limits (mg/L)
24	*P* = −22.79 + 9.99C	0.925	191.25	179.37–215.75
48	*P* = −23.83 + 10.72C	0.941	166.81	159.49–176.55
72	*P* = −28.01 + 12.80C	0.900	154.30	148.36–160.59
96	*P* = −25.87 + 11.96C	0.919	145.24	138.94–151.27

In the regression equation, *P* is the probability unit of mortality and C is the logarithm of the concentration of MBA. R is the regression coefficient.

**Table 2:** Estimated 96 h LC_50_ values, 95% confidence limits and toxicity categories of MBA and some other herbicides to *P. clarkii.*

Herbicides	96 h LC_50_ (mg/L)	95% Confidence Interval (mg/L)	US EPA Toxicity Category	Reference
Sulfometuron	12,174	11,890–12,360	Practically non-toxic	[28]
MBA	145.24	138.9–151.3	Practically non-toxic	This study
2,4-D	185	75–343	Practically non-toxic	[21]
Molinate	14	11–16	Slightly toxic	[27]
Trifluralin	12	11–13	Slightly toxic	[35]
Propanil	7.9	6.8–8.6	Moderately toxic	[27]
Paraquat	1.4	0.5–3	Moderately toxic	[36]
Thiobencarb (for juveniles)	0.47	0.36–0.58	Highly toxic	[37]
Thiobencarb (for adults)	0.20	0.09–0.31	Highly toxic	[37]

MBA: mixture of bensulfuron-methyl and acetochlor.
